# Impact of physician-staffed ground emergency medical services-administered pre-hospital trauma care on in-hospital survival outcomes in Japan

**DOI:** 10.1007/s00068-023-02383-w

**Published:** 2023-11-24

**Authors:** Motohiro Tsuboi, Manabu Hibiya, Hiroyuki Kawaura, Nozomu Seki, Kazuki Hasegawa, Tatsuhiko Hayashi, Kentaro Matsuo, Shintaro Furuya, Yukiko Nakajima, Suguru Hitomi, Kaoru Ogawa, Hajime Suzuki, Daisuke Yamamoto, Masahiro Asami, Saki Sakamoto, Jiro Kamiyama, Yuko Okuda, Kazu Minami, Katsunobu Teshigahara, Masashi Gokita, Koichi Yasaka, Shigemasa Taguchi, Kazuya Kiyota

**Affiliations:** 1 Advanced Emergency and Critical Care Center, Saitama Red Cross Hospital, 1-5, Shintoshin, Chuo-Ku, Saitama, Saitama, 330-8553 Japan; 2 International Cooperation for Disaster Medicine Lab., International Research Institute of Disaster Science (IRIDeS), Tohoku University, 468-1, Aramaki-aza-Aoba-Ku, Sendai, Miyagi, 980-8572 Japan; 3 Teikyo Academic Research Center, Teikyo University, 2-11-1, Kaga, Itabashi-Ku, Tokyo, 173-8605 Japan

**Keywords:** Trauma, Pre-hospital care, Emergency medical service, Doctor car, Survival outcome

## Abstract

**Purpose:**

In Japan, the vehicle used in pre-hospital trauma care systems with physician-staffed ground emergency medical services (GEMS) is referred to as a “doctor car”. Doctor cars are highly mobile physician-staffed GEMS that can provide complex pre-hospital trauma management using various treatment strategies. The number of doctor car operations for patients with severe trauma has increased. Considering facility factors, the association between doctor cars and patient outcomes remains unclear. Therefore, this study aimed to examine the relationship between doctor cars for patients with severe trauma and survival outcomes in Japan.

**Methods:**

A nationwide retrospective cohort study was conducted to compare the impact of the doctor car group with the non-physician-staffed GEMS group on in-hospital survival in adult patients with severe trauma. The data were analyzed using multivariable logistic regression models with generalized estimating equations.

**Results:**

This study included 372,365 patients registered in the Japan Trauma Data Bank between April 2009 and March 2019. Of the 49,144 eligible patients, 2361 and 46,783 were classified into the doctor car and non-physician staffed GEMS groups, respectively. The adjusted odds ratio (OR) for survival was significantly higher in the doctor car group than in the non-physician staffed GEMS group (adjusted OR = 1.228 [95% confidence interval 1.065–1.415]).

**Conclusion:**

Using nationwide data, this novel study suggests that doctor cars improve the in-hospital survival rate of patients with severe trauma in Japan. Therefore, doctor cars could be an option for trauma strategies.

## Introduction

Adequate management of trauma care systems is a critical public health issue. In 2021, the World Health Organization (WHO) reported that the number of trauma deaths worldwide was 4.4 million yearly [1]. Similarly, Japanese demographic statistics for 2021 recorded approximately 40,000 deaths yearly [2]. The trauma care system consists of several components [3]. In particular, time is crucial in the prognosis of patients with severe trauma; therefore, improving the pre-hospital trauma care system, including pre-hospital and transport care, is vital [4–6]. An association between physician-led pre-hospital management and a decrease in in-hospital mortality was reported in Japan [7, 8]. Furthermore, in physician-led pre-hospital management, the use of the Helicopter Emergency Medical Service (HEMS) for patients with severe trauma decreased in-hospital mortality [9–12]. However, Japanese HEMS face mobility constraints in urban areas, limited aircraft for deployment, a lack of nighttime or adverse weather operations, and lengthy dispatch times. Moreover, improving the prognosis of patients with severe trauma injuries who are transported by ground remains an issue [13, 14].

Through non-physician staffed ground emergency medical services (GEMS) in Japan, paramedics provide pre-hospital trauma care via oxygen administration, spinal immobilization, compression hemostasis, and cardiopulmonary resuscitation. Under a doctor's guidance, paramedics are now able to provide advanced first-aid measures for cardiopulmonary arrest, including semi-automatic defibrillation, tracheal intubation, pre-hospital lactated Ringer's solution treatment, and adrenaline administration [7, 15]. However, the procedures that can be performed by Japanese paramedics are limited [16]. Therefore, in Japan, pre-hospital physician intervention is necessary to achieve pre-hospital trauma management based on various treatment strategies [17].

In Japan, the emergency vehicle used in physician-staffed GEMS is called a doctor car. Emergency medical centers in Japan possessed approximately 260 doctor cars in 2021 in pre-hospital trauma care systems [18]. The Japanese doctor car system is not yet standardized. However, according to a report by the Japanese Ministry of Health, Labor and Welfare [19], in many cases, the criteria for requesting the dispatch of a doctor car are established by a prior written agreement between the organization of emergency services and the doctor car base hospital in each region. For example, a request for dispatching a doctor car can be made by the emergency operations center level to the doctor car base hospital for physiologically and anatomically severe injuries, such as high-energy blunt trauma, penetrating trauma, and gunshot wounds, according to the applicable keyword. In contrast, there are other instances wherein emergency operations centers and paramedics can make requests for dispatching a doctor car based on their own judgment, regardless of the request criteria; a doctor car can be then dispatched from the doctor car base hospital. Doctor car emergency physicians can perform advanced procedures such as ultrasonography to locate the bleeding site, airway management (including surgical airway clearance), securing the infusion tract (including the bone marrow tract and infusion management), thoracentesis and chest drain insertion, resuscitative thoracotomies, resuscitative endovascular balloon occlusion of the aorta (REBOA), and drug administration. In addition, information on the patient’s condition and post-hospital treatment strategy can be constantly communicated in advance to the destination hospital [15]. In France, a physician-staffed GEMS intervention for patients with blunt trauma reduced in-hospital 30-day mortality compared with a non-physician staffed GEMS [20]. However, in Japan’s pre-hospital trauma care system, the impact of doctor car interventions on outcomes for patients with severe trauma remains controversial [15, 21, 22]. Because the limitations of being a multicenter study with a hierarchical structure by facility and the need for additional studies with updated data have been identified [23], and regarding in-hospital care, facilities handling more patients with severe trauma yearly had improved patient outcomes [24]; hence, there is a need to consider facility factors in the analysis of doctor cars and patient outcomes in pre-hospital care [24, 25]. Therefore, this study aimed to compare the impact of doctor car interventions with non-physician-staffed GEMS on in-hospital survival of adult patients with severe trauma, using facility factors and updated national data.

## Methods

### Study design and settings

This was a nationwide retrospective cohort study of the impact of doctor cars on in-hospital survival compared with that of a non-physician staffed GEMS for patients with severe trauma with an injury severity score (ISS) of ≥ 16. Anonymized data were collected from the Japan Trauma Data Bank (JTDB), established by The Japanese Association for Acute Medicine and The Japanese Association for the Surgery of Trauma. Overall, 280 major emergency hospitals were included in the JTDB [21].

### Patients

In total, 372,365 patients with trauma were enrolled in the JTDB between April 2009 and March 2019. The inclusion criteria were as follows: (1) ISS ≥ 16, (2) age 15–85 years, (3) a clear injury history, (4) those transported to the hospital from the scene, and (5) a clear means of transport—a doctor car or non-physician staffed GEM [15]. However, the exclusion criteria were as follows: (1) cardiac arrest at the scene (heart rate = 0 was defined as cardiac arrest); (2) Abbreviated injury scale (AIS) = 6 (an unsalvageable condition); and (3) patients with missing variables necessary for analysis.

### Exposure

In this study, the exposure group used doctor cars in pre-hospital care. This included conventional high-standard ambulances with a physician on board and passenger car-type emergency vehicles approved for operation in Japan in April 2008, without a bed for patient transport, dispatching a physician and a nurse to the scene.

### Control

Non-physician staffed GEMS in pre-hospital trauma care was defined as the control group.

### Outcome measures

The outcome was in-hospital survival at discharge.

### Variables

We obtained the following information from JTDB: age, sex, pre-hospital vital signs (systolic blood pressure [SBP], respiratory rate [RR], heart rate [HR], and Japan coma scale [JCS]) [26], season, injury year, injury time (day or night), injury day (weekday or holiday), trauma type (blunt or sharp), pre-hospital time course (injury to emergency department arrival), facility, ISS and the highest score of AIS values for each region of the body, and patient survival status at hospital discharge [7, 15, 24, 27]. The days of injury were defined as weekdays and holidays based on the Japanese calendar. This study aimed to investigate the impact of pre-hospital doctor car interventions on outcomes, as in previous reports; therefore, physiological information post-hospital arrival was not included [15].

### Statistical analyses

For the baseline characteristics of the patients, categorical and continuous data were expressed as n (%) and mean ± standard deviation (SD), respectively, based on a normal distribution. When data did not follow a normal distribution, continuous data were expressed as medians (interquartile range [IQR]). Patient data were classified into doctor car and non-physician staffed GEMS groups. The chi-squared test, Welch's t-test, and Wilcoxon rank sum test were used to compare categorical, continuous volume, and median [IQR] data, respectively, between groups. The analysis was considered significant if the two-sided *p*-value was < 0.05. A sample size calculation was not performed. Data were analyzed using SAS version 9.4 statistic software (SAS Institute, Inc., Cary, NC, USA).

This study used multivariable logistic regression analysis with adjustment for covariates to explain the association between outcomes and doctor cars. Generalized estimating equations (GEE) were applied to the logistic model to account for the hierarchical structure of the data collected from multiple facilities, that is, clustering by the hospital [28–30]. Covariates included age, sex, injury year, season, injury day, injury time, ISS, pre-hospital vital signs (SBP, RR, HR, and JCS), and pre-hospital time course.

Hospital volume (HV) was defined as the annual number of patients hospitalized with severe trauma (ISS ≥ 16) [24]. Subgroup analyses of the relationship between doctor cars and survival compared with non-physician staffed GEMS were conducted for HV ≥ 50 patients/year and HV < 50 patients/year groups [24].

## Results

### Baseline characteristics

**Fig. 1:**
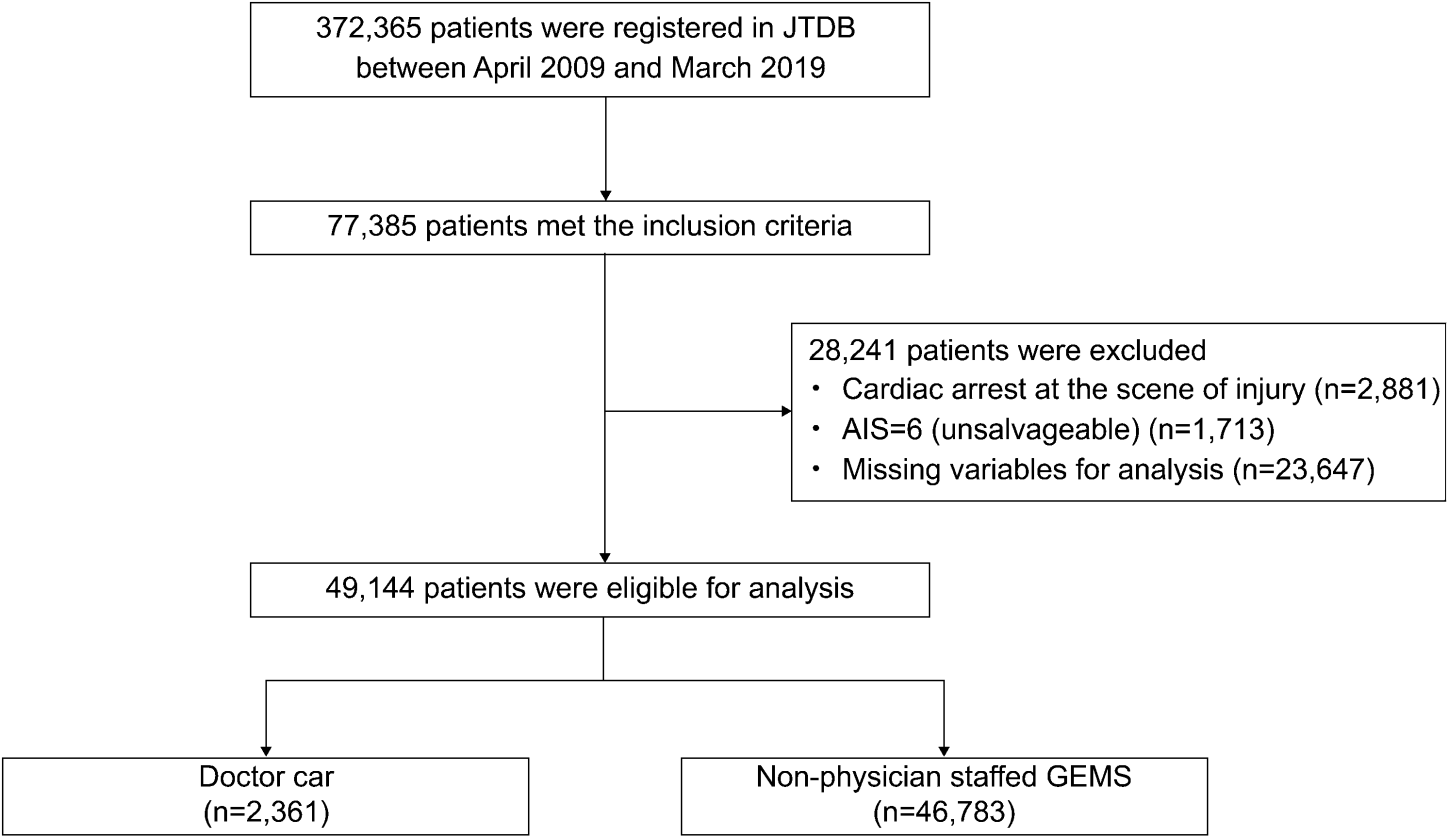
Flow diagram of the process of patient selection. *AIS* Abbreviated Injury Scale, *JTDB* Japan Trauma Data Bank, *GEMS* Ground Emergency Medical Service

The JTDB enrolled 372,365 patients who experienced trauma between April 2009 and March 2019. Figure 1 is a flow diagram of the patient selection process for the 49,144 patients with severe trauma who met the eligibility criteria. Table 1 displays the baseline characteristics of the patients. Overall, 2361 and 46,783 were in the doctor car and non-physician staffed GEMS groups, respectively. The mean age was 52.7 ± 20.5 and 56.5 ± 19.7 years for doctor car and non-physician staffed GEMS groups, respectively (*p* < 0.001). Sex did not differ significantly between both groups (*p* = 0.873). The mean ISS was 27.7 ± 10.4 and 23.5 ± 8.4 in the doctor car and non-physician staffed GEMS groups, respectively, with the doctor car group’s being significantly higher (*p* < 0.001). The mean time from injury to emergency department arrival was 52.3 ± 21.5 min and 40.2 ± 16.1 min in the doctor car and non-physician staffed GEMS groups, respectively, which was longer in the doctor car group (*p* < 0.001). Vital signs at the scene were as follows: RR were 23.9 ± 7.6 times/min and 22.5 ± 6.1 times/min in the doctor car and non-physician staffed GEMS groups, respectively (*p* < 0.001), SBP was 131.5 ± 34.9 mmHg and 136 ± 34.9 mmHg in the doctor car and in the non-physician staffed GEMS groups, respectively (*p* < 0.001). The percentage of conscious patients was significantly lower in the doctor car group, with 444 (18.8%), than in the non-physician staffed GEMS group, with 13,429 (28.7%) (*p* < 0.001). The number of patients according to HV was 1246 (52.8%) in the doctor car group with HV ≥ 50 patients/year and 1115 (47.2%) with HV < 50 patients/year. In the non-physician staffed GEMS group, 19,211 (41.1%) had HV ≥ 50 patients/year, and 27,572 (58.9%) had HV < 50 patients/year.

**Table 1:** Baseline characteristics

Variables	Doctor car group	Non-physician staffed GEMS group	p-value
	(n = 2361)	(n = 46,783)	
Age—year. mean ± SD	52.7 ± 20.5	56.5 ± 19.7	< 0.001^b^
Gender—female—n (%)	667 (28.3)	13,298 (28.4)	0.873^a^
Year of injury—n (%)			
≧2014	1792 (75.9)	32,451 (69.4)	< 0.001^a^
< 2014	569 (24.1)	14,332 (30.6)	–
Season—n (%)			
Spring	579 (24.5)	11,262 (24.1)	0.012^a^
Summer	645 (27.3)	11,670 (24.9)	–
Autumn	650 (27.5)	13,095 (28.0)	–
Winter	487 (20.6)	10,756 (23.0)	–
Day of injury—n (%)			
Holiday	583 (24.7)	15,509 (33.2)	< 0.001^a^
Weekday	1778 (75.3)	31,274 (66.8)	–
Time of injury—n (%)			
Day	1523 (64.5)	27,233 (58.2)	< 0.001^a^
Night	838 (35.5)	19,550 (41.8)	–
Type of trauma—n (%)			
Blunt	2300 (97.4)	46,089 (98.5)	< 0.001^a^
Penetrating	61 (2.6)	694 (1.5)	–
ISS. mean ± SD	27.7 (10.4)	23.5 (8.4)	< 0.001^b^
The highest score of AIS—median [IQR]			
Head	3.0 [0.0, 4.0]	3.0 [0.0, 4.0]	0.160 ^c^
Face	0.0 [0.0, 0.0]	0.0 [0.0, 1.0]	< 0.001^c^
Neck	0.0 [0.0, 0.0]	0.0 [0.0, 0.0]	< 0.001^c^
Chest	0.0 [0.0, 3.0]	3.0 [0.0, 4.0]	< 0.001^c^
Abdomen	0.0 [0.0, 0.0]	0.0 [0.0, 1.0]	< 0.001^c^
Spine	0.0 [0.0, 2.0]	0.0 [0.0, 2.0]	0.070^c^
Upper extremities	0.0 [0.0, 1.0]	0.0 [0.0, 2.0]	< 0.001^c^
Pelvis and lower extremities	0.0 [0.0, 2.0]	0.0 [0.0, 3.0]	< 0.001^c^
Surface	0.0 [0.0, 0.0]	0.0 [0.0, 0.0]	0.363^c^
Vital signs at the scene of injury			
SBP—mmHg. mean ± SD	131.5 (34.9)	136.0 (34.9)	< 0.001^b^
DBP—mmHg. mean ± SD	80.4 (23.0)	80.1 (21.4)	0.524^b^
HR—bpm. mean ± SD	90.2 (23.0)	86.6 (20.6)	< 0.001^b^
RR—times/min. mean ± SD	23.9 (7.6)	22.5 (6.1)	< 0.001^b^
JCS—n (%)			
Clear	444 (18.8)	13,429 (28.7)	< 0.001^a^
I-1, I-2, I-3	797 (33.8)	18,672 (39.9)	–
II-10, II-20, II-30	389 (16.5)	5480 (11.7)	–
III-100, III-200, III-300	731 (31.0)	9202 (19.7)	–
Pre-hospital time course			
Injury to ED arrival—min. mean ± SD	52.3 (21.5)	40.2 (16.1)	< 0.001^b^
Hospital Volume (HV)—n (%)			
HV < 50 patients/year	1115 (47.2)	27,572 (58.9)	< 0.001^a^
HV ≧ 50 patients/year	1246 (52.8)	19,211 (41.1)	–
Outcome on discharge from hospital—n (%)			
Death	352 (14.9)	5341 (11.4)	< 0.001^a^
Survival	2009 (85.1)	41,442 (88.6)	–

Categorical data were expressed as n (%), and continuous data were expressed as mean ± sd, based on a normal distribution. When data did not follow a normal distribution, continuous data were expressed as medians (interquartile range [IQR])

*AIS* Abbreviated Injury Scale, *ED* emergency department, *GEMS* Ground Emergency Medical Services, *HR* heart rate, *HV* hospital volume, *IQR* interquartile range, *ISS* Injury Severity Score, *JCS* Japan Coma Scale [26], *RR* respiratory rate, *SBP* systolic blood pressure, *SD* standard deviation

^a^χ-squared test

^b^Welch’s t-test

^c^Wilcoxon rank sum test

### Impact of the doctor car on in-hospital survival considering facility factors in Japan

Table 2 presents the results of the adjusted ORs for doctor car and in-hospital survival in multivariable logistic regression analysis using GEE. Adult patients with severe trauma, as defined by ISS, had a significant improvement in survival in the doctor car group than in the non-physician staffed GEMS group (adjusted OR 1.228 (95% confidence interval [CI] 1.065–1.415; *p* = 0.005)).

**Table 2:** Adjusted OR for in-hospital survival for each variable in multivariable logistic regression analysis with GEE applied

Variables	Reference	Adjusted OR	95% CI		p-value
Transporter [doctor car]	Non-physician staffed GEMS	1.228	1.065	1.415	0.005
Age	Per 1	0.964	0.962	0.966	< 0.0001
Male	Female	0.857	0.798	0.920	< 0.0001
Year of injury [≧ 2014]	< 2014	1.446	1.351	1.547	< 0.0001
Time of injury [Night]	Day	1.308	1.223	1.399	< 0.0001
Season [Summer]	Spring	1.065	0.973	1.166	0.172
Season [Autumn]	Spring	1.126	1.031	1.230	0.009
Season [Winter]	Spring	1.216	1.107	1.337	< 0.0001
Day of injury [Holiday]	Weekday	0.997	0.931	1.068	0.935
Type of trauma [sharp]	Blunt	0.867	0.657	1.143	0.311
Pre-hospital time course	Per 1	1.003	1.000	1.005	0.025
ISS	Per 1	0.944	0.941	0.948	< 0.0001
SBP [< 90 mmHg]	≧ 90 mmHg	0.742	0.674	0.817	< 0.0001
HR [> 100 bpm]	≦ 100 bpm	0.694	0.646	0.746	< 0.0001
RR [≦ 12 times/min or ≧ 20 times/min]	12 times/min < RR < 20 times/min	0.767	0.708	0.831	< 0.0001
JCS [I-1, I-2, I-3]	0 (clear)	0.453	0.398	0.515	< 0.0001
JCS [II-10, II-20, II-30]	0 (clear)	0.229	0.199	0.265	< 0.0001
JCS [III-100, III-200, III-300]	0 (clear)	0.053	0.046	0.060	< 0.0001

*CI* confidence interval, *HR* heart rate, *ISS* Injury Severity Score, *JCS* Japan Coma Scale [26], *GEE* generalized estimating equation, *OR* odds ratio, *RR* respiratory rate, *SBP* systolic blood pressure

Furthermore, in the HV subgroup, the relationship between the doctor car group and in-hospital survival was examined using multivariable logistic regression analysis with GEE. In facilities with HV ≥ 50 patients/year, the doctor car group had significantly improved in-hospital survival than the non-physician-staffed GEMS group (adjusted OR = 1.288 (95% [CI] 1.051–1.579; *p* = 0.015)). Conversely, in facilities with HV of < 50 patients/year, the relationship between doctor car use and in-hospital survival was not significant (adjusted OR 1.163 (95% [CI] 0.950–1.424; *p* = 0.143)) (Table 3).

**Table 3:** Relationship between doctor car and in-hospital survival in HV subgroups

Subgroup		Adjusted OR	95% CI		p-value
Over all	(n = 49,144)	1.228	1.065	1.415	0.005
HV ≧ 50 patients/year	(n = 20,456)	1.288	1.051	1.579	0.015
HV < 50 patients/year	(n = 28,687)	1.163	0.950	1.424	0.143

*CI* confidence interval, *HV* hospital volume, *OR* odds ratio

## Discussion

This study was the first to use national data to demonstrate that using doctor cars for patients with severe trauma in Japan's pre-hospital trauma care system significantly improved in-hospital survival rates than using conventional, non-physician staffed GEMS. Interestingly, as shown in Table 1, the initial univariate analysis showed that the survival of the doctor car group was poorer when compared to that of the non-physician staffed GEMS group. This finding arose because the doctor car group was more likely to transport more critically ill patients compared to the non-physician staffed GEMS group. However, subsequent multivariable logistic regression analysis adjusted for facility cluster and covariates, such as physiological and anatomical severity revealed that the doctor car group had a significantly higher adjusted OR for in-hospital survival than the non-physician-staffed GEMS group (Table 2). The addition of analyses accounting for hierarchical facility factors to increase internal validity was a strength of this study and was consistent with previous reports elucidating a trend toward improved patient outcomes with doctor-car interventions in patients with severe trauma [15, 22]. The HEMS and doctor cars are vital components of Japan's physician-led pre-hospital trauma care system. The advantages of the HEMS include on-site deployment of medical staff and rapid transport from the scene to the destination hospital. Contrarily, the treatment is performed on-site and is limited during transport owing to safety considerations in Japan [12]. In contrast, doctor car services can conduct seamless activities, including primary surveys and resuscitation, during transportation from the scene to the hospital. Thus, the greatest advantage of doctor car services is that the doctor car can establish an effective pre-hospital trauma strategy by simultaneously transporting and treating the patient [23]. The doctor car may increase pre-hospital time, but the treatment provided during transport may contribute to improved trauma patient outcomes.

Furthermore, Table 3 reveals that the facilities with HV ≥ 50 patients/year had significantly larger adjusted OR for doctor car interventions on in-hospital survival. In the trauma care system, previous studies demonstrated that in in-hospital care, the number of severe trauma patients per facility yearly was associated with reduced in-hospital mortality [24, 25]. This may be because high-volume centers have trauma care resources and systems to improve patient outcomes compared to low-volume centers [31]. However, our findings indicate that the operation of doctor cars in pre-hospital trauma care also suggested that more effective outcomes could be achieved at base hospitals of doctor cars that are proficient in trauma care. This result quantitatively supports a previous report [24] that inferred the possible influence of a comprehensive trauma strategy, including pre-hospital care, as the basis for improved outcomes for trauma patients in high-volume centers. Emergency physicians at doctor car-based hospitals with expertise in trauma care could promptly and accurately identify the severity of the patient's condition before arrival at the hospital, assess treatment priorities, establish the treatment strategy from the injury scene to in-hospital care, provide early intervention, and share this information with the destination hospital, which potentially affects the in-hospital survival rate of patients with severe trauma injury.

This study has several limitations. First, the study included numerous missing values in the exclusion criteria, and the possibility that the effect of doctor cars on the outcome was over-or underestimated could not be ignored. Second, it did not adjust for confounding factors related to the region. Pre-hospital trauma care systems in Japan are established on a regional basis; thus, pre-hospital trauma care systems could differ [25]. Nonetheless, we minimized this effect by conducting an intra-institutional correlation analysis. Furthermore, the generalizability of our findings is limited by the differences in the procedures available to paramedics and the significance of pre-hospital physicians in pre-hospital care, depending on the culture and system of the country. Third, severe trauma was defined as an ISS ≥ 16 based on earlier studies and not by multiple traumas or physiologic severity. Therefore, a possibility of sampling bias among the patients with severe trauma included in this study exists, as opposed to those assumed to be the population. JTDB registration is based on medical records entered by trained inputters; however, errors made in data input may have occurred as chance errors. The impact of chance errors on the study outcomes was small because this was a multicenter study with a large sample size. Moreover, as a retrospective observational study, unmeasured confounders could not be eliminated. However, in the Japanese pre-hospital trauma care system, conducting a randomized controlled trial is ethically difficult because the most severely injured patients are treated by HEMS or doctor cars. Fourth, although the association between doctor cars and patient outcomes in severe trauma was distinct, JTDB information was inadequate, making it impossible to distinguish which treatment strategies affected patient outcomes for specific conditions. Furthermore, although HEMS has a data collection and analysis system [32], a high-quality database unique to doctor cars does not yet exist in Japan. Owing to the lack of evidence, no standardized manuals or guidelines for doctor cars are available, and they have been operated in various ways by each region and medical institution. To ensure efficient and effective doctor car operations with limited human resources in the future, the collection and analysis of high-quality doctor car case studies are crucial to conduct research that considers appropriate patient management according to patients’ conditions, timeframes, and medical institutions.

## Conclusion

This novel study suggests that using doctor cars for patients with severe trauma was associated with improved in-hospital survival compared to non-physician staffed GEMS using national data from Japan. In addition, the study suggests that using doctor cars in pre-hospital trauma care potentially results in more effective outcomes in highly experienced trauma care facilities. Doctor cars can be used for trauma treatment strategies; however, further research and collection of high-quality pre-hospital care data, including condition-specific patient management, time frames and institutions considered, and specific treatment strategies, are needed for high-quality doctor car operations.

## Data Availability

The datasets generated and/or analyzed in the current study are not publicly available due to the need for approval from JTCR.
